# Notes and News

**Published:** 1895-05-04

**Authors:** 


### NOTES AND NEWS.

(COLLECTED AND COMMUNICATED.)

HONG KONG is again in danger of the plague. It has attacked Macao, and traffic between the two places has so far been unrestricted by the authorities.

THE Russian Education Department has decided that female students shall be admitted to the medical faculties of all the Universities of the Empire.

THE Metropolitan Hospital Saturday Fund has formed ambulance classes, and now issues boxes containing all that is necessary for first aid to the wounded.

THE festival banquet of the Royal Eye Hospital, Southwark, will be held on Tuesday, June 18th, at the Whitehall Rooms, Hotel Metropole, Mr. R. K. Causton, M.P., Junior Lord of the Treasury, in the chair.

THE Marquis of Camden will open a bazaar at the Athenæum, Camden Town, in aid of the North-West London Hospital, Kentish Town Road, on Monday, May 13th, at three o'clock. Baron Hirsch has forwarded an additional donation of £500 to the secretary of the North-West London Hospital.

H.R.H. THE DUCHESS OF TECK visited Derby on Wednesday, April 17th, for the purpose of opening a bazaar to raise funds for the new Royal Infirmary. This splendid building has been erected at a cost of about £90,000, of which £18,000 was contributed by the late Mr. George Henry Strutt. The furnishing of the building will necessitate an additional outlay of £4,000, and to collect the money for this purpose the bazaar was inaugurated. Her Royal Highness expressed great admiration for the arrangements and appeared very satisfied with the premises, which may be considered as almost perfect, and on which a vast amount of time and trouble have been expended. There seems no doubt that the bazaar was a distinct success, seeing that £1,500 was taken in two days. We hope to learn later that a considerably larger sum than this was received, and that the profits will reach the required amount.

THE festival dinner of the Royal Medical Benevolent College is announced to take place on Wednesday, May 5th, at Queen's Hall, Langham Place. The chair will be taken by the Right Hon. A. J. Balfour, M.P.

THE Marchioness of Dufferin and Ava, C.I., last Tuesday paid a visit to the Chelsea Hospital for Women, accompanied by her daughter, Lady Hermoine Blackwood. Lady Dufferin is well known as the foundress of the "National Association for Supplying Medical Relief to Women of India," more familiarly known as the "Countess of Dufferin Fund." The association, dnring its ten years' existence, has built 70 hospitals in India; given employment to over 100 qualified medical women, not counting nurses and midwives, working under the fund; and has afforded medical relief to over three million women and children.

MR. HERBERT H. JENNINGS has been appointed to the post of secretary at the Chelsea Hospital for Women, in the place of Mr. A. C. Davis, resigned. Mr. Jennings has had eight years' experience as assistant secretary at Charing Cross Hospital, and we congratulate the Chelsea Hospital authorities upon their choice. It is extremely important that such responsible appointments should be filled by those who have been trained to the work, and it is satisfactory to see that hospital committees are becoming increasingly alive to the advisability of selecting for their secretaries gentlemen of proved ability in the duties of hospital administration.

IT appears that we are not yet sufficiently alive to the dangers that attend the consumption of our daily food. Milk is a much to be suspected part of our diet, owing principally to its liability to convey contagion. In the United States the subject of the conveyance of tuberculosis by milk has been carefully gone into, and one medical journal deduces from the evidence forthcoming that 3 per cent. of all milk supplied to large cities contains sufficient bacilli to prove infectious. Some medical testimony gives a large percentage of victims. Whether the facts are over-estimated or no, it would seem wise to boil all milk used in our households.

THOSE who believe in the health-giving powers of Buxton have always found it difficult to give an explanation of the benefit derived from the use of Buxton waters by rheumatic persons. It is now said that argon, the recently-discovered elementary gas, has much to do with the matter. There is, in fact, just now a sort of "boom" in argon. This gas, although associated with the nitrogen of the atmosphere, is not combined with it, any more than nitrogen is combined with the atmospheric oxygen, and is apparently almost incapable of combination, unless with a still more recently-discovered elementary gas which has been found to be blended with it, and it is now said that, having a solubility in water greater than nitrogen and equal to oxygen, argon would more readily penetrate, or be absorbed by, the skin or other membranes of the body, and, either alone or with other gases, be enabled to act upon, or absorb, or carry away morbid depositions. All this is very interesting, and to those who like to hide their ignorance in many words is no doubt satisfactory; we cannot say, however, that it does much to advance the explanation of the remarkable action of the nitrogenous water of Buxton on the conditions and results of rheumatic, gouty, or fibroid diseases.

